# Evaluation of lymphatic vessel dilatations by anterior segment swept-source optical coherence tomography: case report

**DOI:** 10.1186/s12886-017-0588-y

**Published:** 2017-10-23

**Authors:** Eva Volek, Jeannette Toth, Zoltan Zsolt Nagy, Miklos Schneider

**Affiliations:** 1 St. Lazarus Hospital, Salgotarjan, Hungary; 2 2nd Department of Pathology, Semmelweis University, Budapest, Hungary; 3 Faculty of Medicine Department of Ophthalmology, Semmelweis University, Budapest, Hungary

**Keywords:** Swept-source OCT, Conjunctival lymphangiectasia, Virchow-Robin space

## Abstract

**Background:**

Conjunctival lymphangiectasia is a rare condition presumably caused by the obstruction of lymphatic channels or by an abnormal connection between conjunctival lymphatic and blood vessels. Diagnosis is based on clinical appearance and histology. We report a case of conjunctival lymphangiectasia in which anterior segment optical coherence tomography (OCT) was used to assist the diagnosis and the planning of the biopsy location.

**Case presentation:**

A 31-year-old woman was referred with repeated episodes of conjunctival “hemorrhages” and chemosis with extended recovery periods over the last months. Other symptoms were dryness, redness, burning sensation and itching. Photo documentation, anterior segment OCT, ultrasound, computer tomography (CT) and magnetic resonance imaging (MRI) of the brain were performed. MRI revealed dilated atypical Virchow-Robin space (VRS). Conjunctival biopsy was taken and the location of the biopsy was selected based on OCT findings.

Based on the clinical appearance we suspected the case to be conjunctival lymphangiectasia or lymphangioma. Histology and immunhistochemistry confirmed the diagnosis of conjunctival lymphangiectasia.

**Conclusions:**

Anterior segment OCT is a non-invasive tool, useful in the evaluation of conjunctival lesions and planning surgery.

## Background

Conjunctival lymphatic vessel dilatation also known as conjunctival lymphangiectasia is a rare condition which is presumably caused by the obstruction of lymphatic channels or by the abnormal connection between conjunctival lymphatic and blood vessels [1–3]. Etiology is often unknown, it may be caused by trauma, inflammation, neoplasia or it can occur as complication of ocular interventions. It exists in two different forms: (1) as a diffuse enlargement of lymphatic vessels with the clinical appearance of chemosis, and (2) as focally dilated lymphatics that manifests as series of cysts (“string of pearls”) or sausage-shaped clear-walled channels. The latter may become filled with blood (hemorrhagic lymphangiectasia). The condition is usually unilateral, non-congenital unless associated with Turner, and Klippel-Trenaunay-Weber syndromes [1, 4, 5]. It is frequently asymptomatic and found accidentally on routine eye examinations [6]. Symptoms may be red eye, foreign body sensation, dryness, irritation, chemosis, epiphora, blurred vision, and pain [2, 7]. Differential diagnosis includes epithelial inclusion cyst, cystic conjunctival nevus, conjunctival lymphangioma, conjunctivochalasis, allergic conjunctivitis, telangiectasia and neoplasia [3, 7]. Diagnosis is based on clinical appearance and histology. Although lymphangiectasia can resolve spontaneously [8] in symptomatic cases topical steroids, surgical intervention (subconjunctival injection of Bevacizumab, excision, liquid nitrogen cryotherapy, or high-frequency radio wave electrosurgery) may be required [2, 7, 9–11].

## Case presentation

A 31-year-old woman reported with more episodes of conjunctival hemorrhages and chemosis with extended healing periods over the last months. Other symptoms were dryness, redness, burning sensation and itching. She was followed over the last 15 years by another department because of dilatation of conjunctival blood vessels in the right eye. Additional examinations were not performed. Medical history of the patient was unremarkable, there was no history of ocular surgery, trauma or any other ocular or general diseases. She had no family history of any significant disease.

**Fig. 1:**
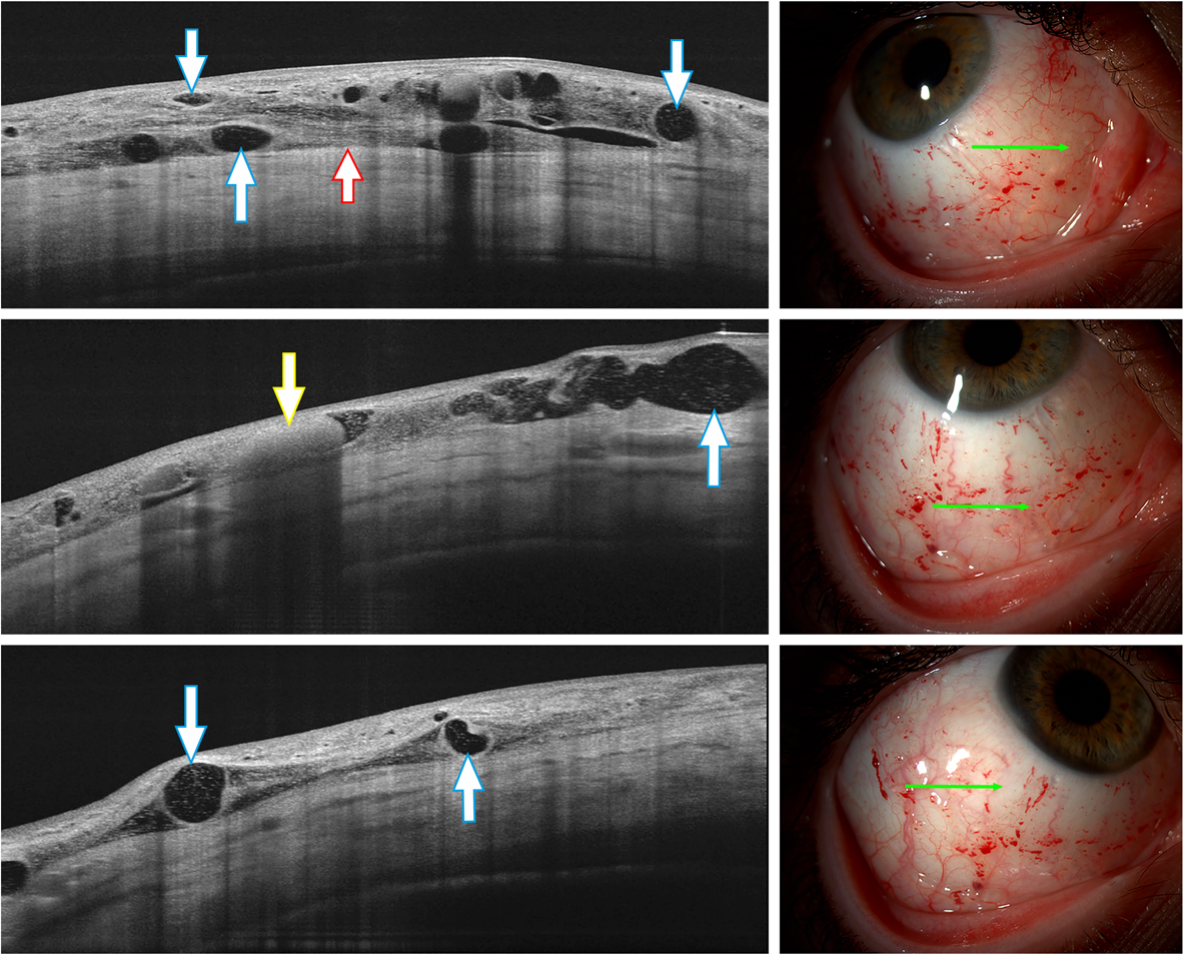
Slit lamp photographs (right side): Diffuse chemosis, dilated, tortuous conjunctival vessels and hemorrhages with ‘dot/blot’ configuration affecting the temporal, nasal and inferior bulbar conjunctiva of the right eye. Green arrows showing the locations where OCT scans were taken. Swept-source OCT scans (left side): Subconjunctival hemorrhages appear as hyperreflective areas (yellow arrow). Dilated lymphatic vessels are shown as hyporeflective spaces (blue arrows) with widely varying calibers, OCT revealed clear fluid-filled spaces demarcated by septae within the elevated conjunctiva. The sclero-conjunctival interface is also easily visible in high resolution (red arrow)

Visual acuity was 20/20 with myopic correction in both eyes. Slit lamp biomicroscopy revealed diffuse chemosis, dilated, tortuous blood-filled conjunctival vessels, affecting the temporal, nasal and inferior quadrant of the right eye (Fig. 1.). The lesion was mobile and not fixed to the underlying sclera. Anterior segment of the left eye showed no pathologies, posterior segment of both eyes was normal.

**Fig. 2:**
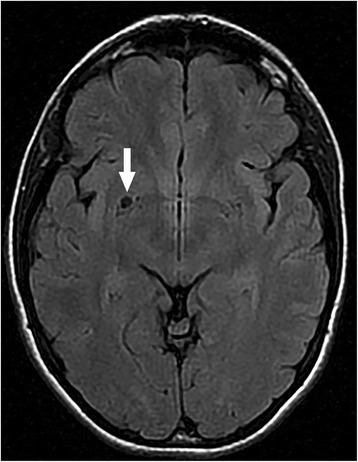
Magnetic resonance imaging (MRI) scan. Dilated atypical Virchow-Robin space (VRS) under the basal ganglia on the right side is visible (white arrow)

Ophthalmic ultrasound and cranial computed tomography (CT) did not reveal any abnormalities. Magnetic resonance imaging (MRI) disclosed dilated atypical Virchow-Robin space (VRS) on the right side (side of the dilated lymphatic vessels). The dilated VRS was round, 5–6 mm in diameter and located under the basal ganglia (Fig. 2.).

Based on the clinical appearance our differential diagnosis was conjunctival lymphangiectasia or possibly lymphangioma. Swept-source OCT (DRI OCT-1 Model Triton (plus), Topcon, Tokyo, Japan) scans were taken of both eyes using the anterior segment (AS) module of the device. On the right side subconjunctival hemorrhages appeared as hyperreflective areas, while the dilated lymphatic vessels were shown as hyporeflective spaces with widely varying calibers on the OCT images (Fig. 1). OCT also revealed clear fluid-filled spaces demarcated by septae within the elevated conjunctiva. On OCT imaging the dilated calibers of the vessels and the border between the conjunctiva and the sclera was easily visualized in high resolution. It was also easy to determine which areas of the subconjunctiva were the most densely filled with dilated lymphatic vessels. Location for the conjunctival biopsy was selected based on the above OCT findings.

**Fig. 3:**
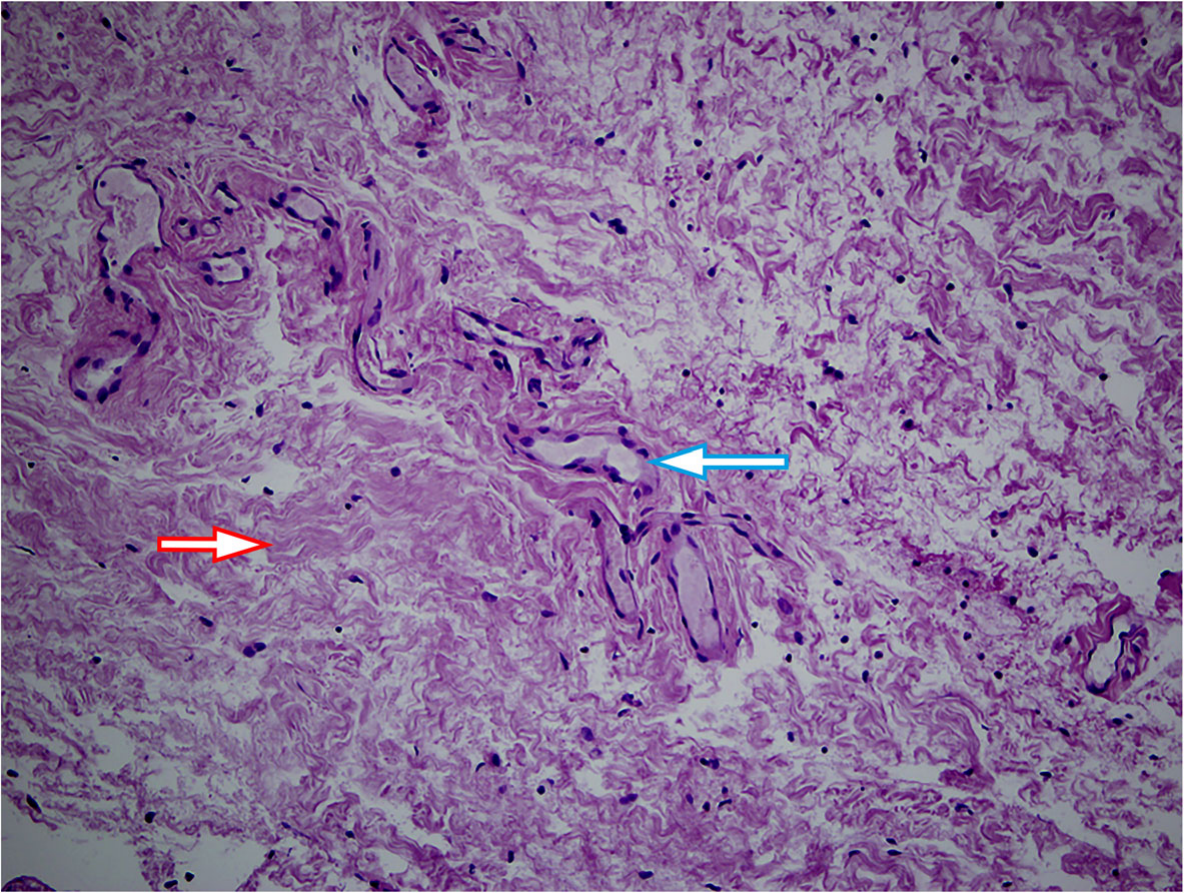
Histology with Hematoxylin and Eosin staining (200× magnification). The sample shows irregularity and thickening of the collagen fibers (red arrow). Between the fibers sparsely dilated lymphatic channels are seen in small groups (blue arrow)

A sample was taken from the subconjunctiva all the way to the scleral surface in the inferonasal area on the right eye under local anaesthesia. Histology with Hematoxylin and Eosin staining (Fig. 3) and immunohistochemistry with D2–40 lymphatic endothelial marker monoclonal antibody were performed (not shown). The sample showed irregularity and thickening of the collagen fibers. Between the fibers we found sparsely dilated lymphatic channels in small groups. Histology confirmed the diagnosis of conjunctival lymphangiectasia.

## Discussion and conclusions

Conjunctival lymphangiectasia is a rare and benign disease. Treatment is only required in symptomatic cases.

On the MRI examination we found atypical VRS on the same side as lymphangiectasia and central nervous system (CNS) lymphoma could be excluded. VRSs are regarded as perivascular spaces containing perforating arteries extending into the brain parenchyma. They have an important role in the homeostasis of cerebral fluids in the CNS. VRS enlargement is possibly correlated with the disturbance of cerebro-spinal fluid (CSF) dynamics. Various disorders such as Alzheimer’s disease, cerebrovascular disease and head trauma are associated with a higher frequency of VRS, but the relevance of VRS frequency to idiopathic normal pressure hydrocephalus (iNPH) is unclear [12]. After consultation with neurologists we believe that the VRS in this case is of lesser significance and is a coincidental finding.

AS-OCT is helpful in establishing an in vivo diagnosis of certain diseases of the anterior segment and in the differential diagnosis of diseases of the ocular surface. It is a non-contact, non-invasive imaging device that provides high resolution, real-time and in situ visualization of tissue microstructure. Disadvantages of the AS-OCT is the limited visibility of the underlying sclera and the difficulty determining the relation between conjunctival lesions and the sclera. Thickening, vascularization and pigmentation of conjunctival lesions can also cause difficulties in imaging the underlying sclera [13]. Additionally, in cases of peripheral conjunctival lesions there may be difficulties in visualizing the area with the device.

In our case anterior segment swept-source OCT was helpful in the diagnosis and in the planning of the biopsy. Determining the optimal sampling site for the biopsy with AS-OCT can result in histology slides with higher density of the abnormalities in question, avoiding poor sample collection, helping the work of pathologists and aiding the accurate diagnosis.

## Abbreviations

AS: OCT anterior segment optical coherence tomography
CSF: Cerebro-spinal fluid
CT: Computer tomography
iNPH: Idiopathic normal pressure hydrocephalus
MRI: Magnetic resonance imaging
OCT: Optical coherence tomography
VRS: Virchow-Robin space
